# Addition of clobazam successfully treating drug resistant seizures in Heidenhain variant Creutzfeldt Jakob disease: A case report

**DOI:** 10.1016/j.ebr.2023.100585

**Published:** 2023-01-12

**Authors:** Jason M. Maille, Sebastian S. Hanna, Darshan N. Shah

**Affiliations:** a Texas A&M University College of Pharmacy, 59 Reynolds Medical Building, College Station, TX 77843, USA; b University of Vermont Larner College of Medicine, 89 Beaumont Ave, Burlington, VT 05405, USA; c Seton Brain and Spine Institute Neurology, 1601 Trinity St #804, Austin, TX 78701, USA

**Keywords:** Creutzfeldt Jakob disease, Seizure, Benzodiazepine, Clobazam, RT-QuIC, Heidenhain variant

## Abstract

Creutzfeldt Jakob Disease (CJD) is a rapidly progressive and fatal neurodegenerative disease that is uncommonly accompanied with seizures. In this case report, we describe a 63-year-old male patient who presented with a 3-week history of visual disturbances and clonic movement of his left arm. Additionally, the patient was reported to have developed erratic behaviors along with insomnia during this period. An EEG showed 4 electrographic seizures of bilateral temporo-occipital onset characterized by 1.5 Hz periodic discharges, lasting 2–13 min. Levetiracetam was started and titrated to the maximal dose however seizures continued so lacosamide and clonazepam were initiated. Despite these aggressive treatments, seizures continued, and oral clobazam 5 mg BID replaced clonazepam. Continued electrographic seizures warranted an increase in clobazam to 10 mg BID after which the seizures stopped; of note, lateralized periodic discharges (LPDs) remained. The patient’s symptoms were consistent with the Heidenhain variant, along with probable CJD due to positive RT-QuIC assay, positive 14-3-3 protein, MRI FLAIR hyperintensities, and EEG findings. Although the patient passed away 3 weeks following admission as a result of CJD, we propose that there may be clinical benefit in the use of clobazam in suspected CJD patients presenting with seizures, and its use merits further investigation.

## Highlights

• Creutzfeldt-Jakob Disease (CJD) rarely presents with seizures, but are often resistant to anti-epileptic drugs.
• Patient presented with visual disturbances that remained predominant during clinical course consistent with Heidenhain variant CJD (HvCJD)
• Recorded seizures responded to clobazam, levetiracetam, and lacosamide.

## Introduction

Creutzfeldt-Jakob disease (CJD) was first described in 1920 by the German neuropathologist Hans Gerhard Creutzfeldt [1]. It is a rare progressive and fatal neurodegenerative disease caused by prions, or transmissible proteinaceous particles, and it occurs in about 1 per million people worldwide.

Cardinal symptoms of the disease include rapidly progressive dementia, cerebellar and extrapyramidal signs, myoclonus, and visual symptoms. Seizures, a rare manifestation of CJD, may present in the later stages of the disease [2]. A review article found that epileptic seizures occurred in 3 % of CJD patients at the onset of disease, and in 12 % during the entire clinical course [3]. Most patients diagnosed with CJD die within a year of symptom onset [4].

Periodic Sharp Wave Complexes (PSWC), typically biphasic or triphasic in appearance, are widely accepted as the most characteristic EEG abnormality in CJD [3], [5], [6]. Brain MRIs with fluid-attenuated inversion recovery (FLAIR) or diffusion-weighed imaging (DWI) showing hyperintensities can further support a CJD diagnosis [6], [7]. Positive 14-3-3 protein and highly elevated total-Tau (*t*-Tau) protein are typically observed in CJD [6]. RT-QuIC assay is a more accurate diagnostic test with a specificity nearing 100 % [8] and is the gold standard for CJD diagnosis in the absence of autopsy [6], but often can take weeks for results.

Seizure evaluation and control remains particularly problematic in patients with a rapidly deteriorating neurological disease such as CJD. Patients present with altered mentation or other neuropsychiatric symptoms, and it is often unclear if such symptoms are secondary to the underlying disease or seizures/status epilepticus. Long term EEG monitoring is essential in the ongoing evaluation of such patients and aggressive seizure management with anticonvulsants is paramount in reducing/limiting neuronal cytotoxicity resulting from ongoing seizures. Treatment also removes seizures as a confounding factor in the patient’s presentation.

Various attempts to treat seizures in CJD patients have been documented with varying success. Diazepam, a drug in the benzodiazepine class, has previously been reported to suppress epileptiform discharges in patients in CJD without improvement in clinical status [9]. Valproic acid and clonazepam have been reported to control myoclonic jerks with some slight improvement reported [10]. Another case report described unsuccessful treatment of a patient’s seizures with levetiracetam, lacosamide, valproic acid, perampanel, and phenobarbital [11].

## Case report

### Hospital admission

The patient was a 63-year-old Caucasian male with a past medical history significant for anxiety, depression, bipolar disorder, and type 2 diabetes mellitus. Three weeks prior to admission, the patient was traveling and experienced visual disturbances while driving, including macropsia, where objects appeared abnormally large in the middle of his vision. Upon returning home, his vision remained altered, and he began to have jerking movements in his dominant left arm. He later began experiencing insomnia and waking in the middle of the night feeling as if he had to leave his home to go somewhere. He disassembled a working lamp and urinated on a pile of towels as if it were a toilet. Considering the strange behavior, the spouse became increasingly worried and brought the patient to the ED.

Initial serologic testing for altered mental status including ammonia, toxicology screening, TSH, B12 and folate was negative. HIV, EBV, and testing for SLE was also negative. BG levels ranged from 136 to 273 mg/dL. No clear infectious etiology contributing to the patient’s encephalopathy was found. Admission brain MRI showed no acute abnormalities and mild chronic small vessel disease.

### Seizures and management

**Fig. 1:**
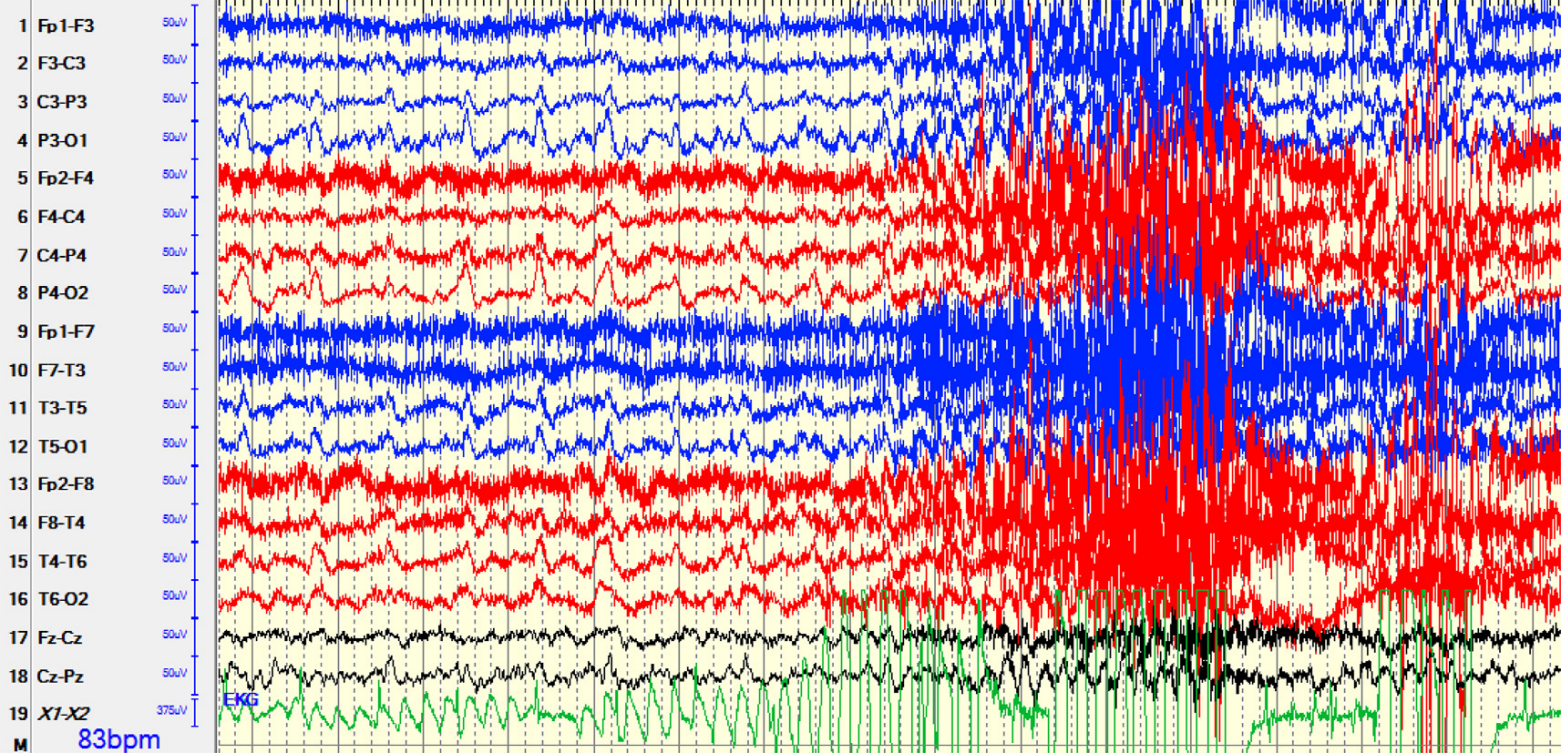
Recorded Activity on LTEEM – Admission Day 3. EEG shows focal onset in the bilateral temporo-occipital lobes characterized by 1.5 Hz periodic discharges evolving into rhythmic 4 Hz delta activity seen predominantly in the bilateral occipital lobes.

Long Term EEG Monitoring (LTEEM) was started on admission day 2 and initially showed right occipital seizures for which levetiracetam 1 g IV BID was initiated. The seizures continued through the afternoon and levetiracetam was increased to 2 g IV BID; a lacosamide loading dose of 200 mg IV was given followed by maintenance of 100 mg IV BID; clonazepam 0.5 mg oral BID was also started. LTEEM revealed 4 electrographic seizures of bilateral temporo-occipital onset characterized by 1.5 Hz periodic discharges, lasting 2–13 min, diffuse slowing and 8 Hz posterior-dominant rhythm. On admission day 3, EEG readings showed seizures with a focal onset in the bilateral temporo-occipital lobes characterized by 1.5 Hz periodic discharges, evolving into rhythmic 4 Hz delta activity seen predominantly in the bilateral occipital lobes (Fig. 1). The location of the seizures clinically fit with the patient's presentation of visual disturbances.

**Fig. 2:**
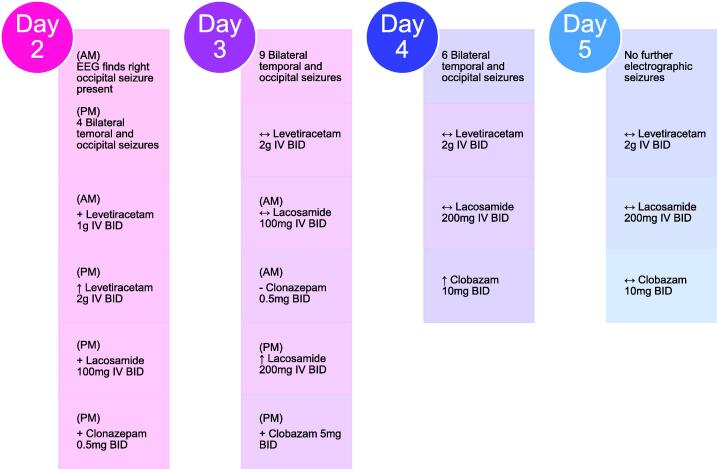
The antiseizure medication administration record following the first 2–5 days of admission. Therapy from day 5 was continued until patient’s death on day 24. + = Addition of medication | ↑ = Dose increase | - = Removal of medication | ↔ = Continuation of medication and dose.

Due to drug resistant seizures on admission day 3, lacosamide was further increased to 200 mg IV BID. Clonazepam was discontinued and clobazam 5 mg oral BID was started. LTEEM the following day showed 9 electrographic seizures of bilateral temporo-occipital onset characterized by 1.5 Hz periodic discharges, lasting 2–13 min, and bilateral temporo-occipital lateralized periodic discharges (LPDs). Clobazam was increased to 10 mg oral BID. Notably, after an additional day on the combination regimen of levetiracetam 2 g IV BID, lacosamide 200 mg IV BID, and clobazam oral 10 mg BID, the patient had no further electrographic seizures on day 5 (Fig. 2).

**Fig. 3:**
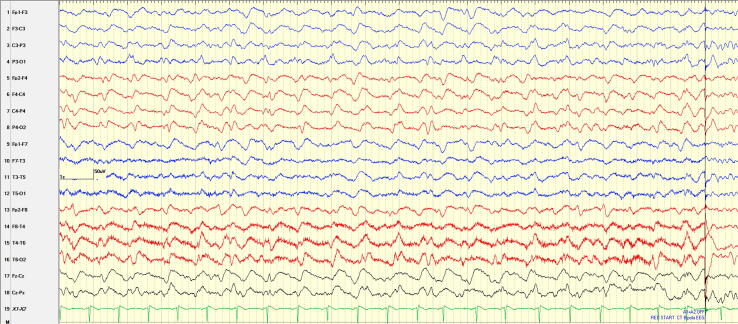
Recorded Activity on LTEEM – Admission Day 14. EEG shows a similar 1.5 Hz rhythmicity with a wider complex and an almost triphasic appearance resembling LPDs.

Because no further seizures were witnessed on day 5, LTEEM was stopped until day 14 to provide the patient scalp rest. During this 9-day period, patient continued to receive anticonvulsants including clobazam. LTEEM was resumed on day 14 to monitor disease progression and antiseizure medication efficacy (Fig. 3). The findings showed the rhythmicity of discharges remained the same at 1.5 Hz, however the morphology changed to a wider complex with an almost triphasic appearance, overall resembling LPDs. Sleep architecture seen on EEGs support these being LPDs rather than seizures. Clinically the patient’s visual acuity could no longer be assessed. We hypothesize the EEG changes are in part due to treatment of the seizures with anticonvulsants, progressive destruction of neural networks, and the typical progression expected in CJD as the patient's clinical condition declined.

### Creutzfeldt-Jakob disease diagnosis

The patient continued to decline clinically following the onset of his drug resistant seizures. Neurological examination revealed increased solemness, increased clonic movement of upper extremities, worsening visual status – including new onset left hemineglect, worsening akinesia, decreased motor coordination, and worsening mental status. Brain MRI on admission day 14 demonstrated cortical-based T2 FLAIR hyperintensity and elevated diffusion-weighted signal within the right parietal and posterior temporal region and additionally in the left occipital region. These findings were significantly changed from the previous imaging at admission day two and highly suspicious for CJD [7] or encephalitis.

Cerebrospinal fluid (CSF) testing was negative for autoimmune and viral antibodies. Differential diagnosis was narrowed to antibody-negative autoimmune encephalitis and CJD. A CSF sample was sent to the National Prion Disease Pathology Surveillance Center in Ohio for analysis.

As seronegative autoimmune encephalitis remained in the differential, once daily IV methylprednisolone 1 g and Plasma Exchange (PLEX) every other day was initiated. The patient’s clinical condition briefly improved but then relapsed and his condition worsened. Autoimmune encephalitis was thought to be unlikely at this stage following lack of treatment response. The patient was given a diagnosis of “probable” CJD based on the CDC’s diagnostic criteria (Table 1) [12].

**Table 1:** 2018 Center for Disease Control Diagnostic Criteria for Probable CJD.

**Positive RT-QuIC** or **Rapidly progressing dementia** and at least 2 of the following:	
	**Myoclonus**
	**Visual and cerebellar signs**
	**Pyramidal/extrapyramidal signs**
	**Akinetic mutism**
And at least one of the following:	
	**Atypical EEG**
	**Positive 14**–**3-3 assay in disease duration less than 1 year**
	**MRI (Magnetic Resonance Imaging) positive for FLAIR or DWI**
And **no other significant differential diagnosis**	

**Note.** Bolded criteria are present in patient’s case. Source: Adapted from CDC; CDC does not endorse this table; Diagnostic criteria is available on the agency’s website for no charge.

After 24 days of admission, and 6 weeks after the onset of symptoms, the patient passed away. Postmortem, the CSF sample sent to the national prion center tested positive for RT-QuIC, 14–3-3 protein, and highly elevated *t*-Tau protein further supporting the probable CJD diagnosis [6], [8], [13]. No autopsy was performed per family requests, therefore definite CJD could not be concluded.

The clinical symptoms demonstrated by our patient were typical of the Heidenhain variant of CJD (HvCJD), a subvariant of sporadic CJD, that is characterized by visual disturbance being a leading symptom that persists throughout the course of the disease. The initial presenting symptom of this patient was the inability to operate motor vehicles due to macropsia. During the disease course, he went on to develop rapid deterioration of vision with worsening macropsia, optical hallucinations, and hemineglect. One study looking at medical records of 169 sporadic CJD patients reported that about 20 % met criteria for the Heidenhain variant. [14].

## Discussion

We report a case in which a patient experiences rapidly progressing dementia with drug resistant seizures that died within 6 weeks of symptom onset. He was diagnosed with probable CJD based on the criteria outlined by the Center for Disease Control and Prevention (Table 1) [12]. During his hospitalization, the patient’s seizures did not respond following administration of levetiracetam, lacosamide, or clonazepam. Of note, the patient’s drug resistant seizures appeared to react well when clonazepam was switched to clobazam. After the initiation of clobazam with levetiracetam and lacosamide, seizures decreased in frequency until they stopped within 3 days.

Clobazam is a long-acting benzodiazepine that potentiates GABA, an inhibitory neurotransmitter. Activation of the GABA receptor subtype, GABA-A, results in an increased permeability to chloride ions in neuronal cells which leads to hyperpolarization and decreased neuronal excitability. Clobazam has a long half-life in adults, 36–42 h, which makes it an ideal benzodiazepine (BZD) for long-term seizure management [15]. The long half-life limits clobazam’s onset of action and time to steady state concentration (C_ss_). It typically takes 3 to 4 half-lives to obtain 90 % of C_ss_. With clobazam, that equates to 4.5 to 7 days. Each BID dose of clobazam accumulates before the previous dose exerts one half-life. At 90 % C_ss_, the amount of drug in body tends to remain relatively consistent. Even though a patient may experience symptom relief before steady state, the drug is not exerting its maximal effect.

For the first 48 h after starting clobazam, EEG continued to show ongoing seizures. Within 72 h, prior to achieving clobazam steady state, the seizures stopped. EEG continued to show LPDs which, while epileptogenic and expected in a patient with rapidly progressive neurological disease such as CJD, do not represent ongoing seizures.

There have been studies performed that suggest that the benzodiazepine receptor is preserved in CJD patients. One of these studies compared baseline flash-evoked seizure potentials (FEPs) to flumazenil 5 mg IV, followed by diazepam 10 mg IV administration. The study found that flumazenil potentiated seizures while diazepam reversed the effects induced by flumazenil [16]. To add upon previous FEPs studied, somatosensory cortical evoked potentials (SEPs) were studied in a CJD patient comparing baseline to diazepam IV 10 mg, followed by administration of flumazenil IV 3 mg. They found that seizures were reduced by the diazepam, then potentiated again by the addition of flumazenil [17]. Since flumazenil antagonized and reversed the effects of the benzodiazepine, and considering the results of this report, it is worth noting the effectiveness of benzodiazepines in CJD.

Despite the promising benzodiazepine receptor preservation discovered in these two studies, in practice, BZDs have been used in CJD with mixed results. A case series reported that the BZD, diazepam, suppressed EEG periodic discharges in a CJD patient but enhanced their background activity [18]. In a more recent case report, a patient with probable CJD and focal tonic seizures of the left hand with impaired awareness was given gabapentin 1200 mg/day plus clobazam 20 mg/day with no clinical improvement [19]. Detailed in this report, the patient was trialed with two doses of clonazepam with no effect, then switched to clobazam with some objective improvement after the first three doses were given.

It is worth noting that clonazepam was not given an adequate trial in this patient, only administering two 0.5 mg doses. It is possible that clonazepam may have had a similar effect to clobazam on seizure control after optimizing the dose. Clobazam was selected in this patient because it’s a 1,5 BZD that has a higher affinity for the α_2_ receptor, causing less sedation than 1,4 BZD such as diazepam and clonazepam [20]. Once seizure control was obtained with clobazam, it was felt that it was unnecessary to switch to another BZD.

The success of clobazam in this CJD patient case is promising. Most dosing procedures recommend initiating clobazam at 5–15 mg/day, with slow titration to achieve clinical effect and tolerability. Our patient was started on one 5 mg dose of clobazam, then took 10 mg BID thereafter. Based on our findings, we propose that patients with seizures in the setting of suspected CJD may benefit from aggressive clobazam or BZD treatment and rapid dose optimization as necessary.

## Conclusion

This case report details a patient that presented with hallmark CJD symptoms. He was also found to be experiencing occipital and temporal seizures upon admission. The predominance of visual symptoms throughout disease course was consistent with the Heidenhain variant of sporadic CJD. Seizures were resistant to multiple antiseizure medications. After three doses of clobazam in combination with high dose levetiracetam and lacosamide, seizures appeared to respond and cease. The patient experienced 12 seizures before the first administration of clobazam, and 8 more during its use before the seizures stopped. Due to the seizure control reported in this case, benzodiazepine receptors may be preserved in CJD patients, suggesting that clobazam or other benzodiazepines may be efficacious in CJD-affected individuals. Further cases may validate the efficacy of clobazam and other benzodiazepines. Initiation of clobazam should be an early therapeutic consideration for suspected CJD patients presenting with seizures. After initiation, we suggest aggressive upward dose titration due to the rapid progression of CJD, and the long half-life of clobazam requiring multiple days to exert maximal effect.

## Ethical statement

This is a case report, and consent for treatment and publication was obtained according to our institution’s standards.

**Originality and plagiarism:** Jason Maille, Sebastian Hanna, Darshan Shah.

**Data Access and Retention:** Jason Maille, Sebastian Hanna, Darshan Shah.

## Declaration of Competing Interest

The authors declare that they have no known competing financial interests or personal relationships that could have appeared to influence the work reported in this paper.
